# Activation of the Transcription of *BrGA20ox3* by a BrTCP21 Transcription Factor Is Associated with Gibberellin-Delayed Leaf Senescence in Chinese Flowering Cabbage during Storage

**DOI:** 10.3390/ijms20163860

**Published:** 2019-08-08

**Authors:** Xian-mei Xiao, Yan-mei Xu, Ze-xiang Zeng, Xiao-li Tan, Zong-li Liu, Jian-wen Chen, Xin-guo Su, Jian-ye Chen

**Affiliations:** 1 State Key Laboratory for Conservation and Utilization of Subtropical Agro-bioresources/Guangdong Provincial Key Laboratory of Postharvest Science of Fruits and Vegetables/Key Laboratory of Biology and Genetic Improvement of Horticultural Crops (South China) of Ministry of Agriculture/Engineering Research Center of Southern Horticultural Products Preservation, Ministry of Education, College of Horticulture, South China Agricultural University, Guangzhou 510642, China; 2 Scientific Observing and Experimental Station of Crop Cultivation in South China, Ministry of Agriculture, College of Agriculture, South China Agricultural University, Guangzhou 510642, China; 3 Department of Food Science, Guangdong Food and Drug Vocational College, Guangzhou 510520, China

**Keywords:** Chinese flowering cabbage, GA, leaf senescence, transcriptional activation

## Abstract

Several lines of evidence have implicated the involvement of the phytohormone gibberellin (GA) in modulating leaf senescence in plants. However, upstream transcription factors (TFs) that regulate GA biosynthesis in association with GA-mediated leaf senescence remain elusive. In the current study, we report the possible involvement of a TEOSINTE BRANCHED1/CYCLOIDEA/PCF (TCP) TF BrTCP21 in GA-delayed leaf senescence in Chinese flowering cabbage. Exogenous GA_3_ treatment maintained a higher value of maximum PSII quantum yield (Fv/Fm) and total chlorophyll content, accompanied by the repression of the expression of senescence-associated genes and chlorophyll catabolic genes, which led to the delay of leaf senescence. A class I member of TCP TFs BrTCP21, was further isolated and characterized. The transcript level of *BrTCP21* was low in senescing leaves, and decreased following leaf senescence, while GA_3_ could keep a higher expression level of *BrTCP21*. BrTCP21 was further found to be a nuclear protein and exhibit trans-activation ability through transient-expression analysis in tobacco leaves. Intriguingly, the electrophoretic mobility shift assay (EMSA) and transient expression assay illustrated that BrTCP21 bound to the promoter region of a GA biosynthetic gene *BrGA20ox3*, and activated its transcription. Collectively, these observations reveal that BrTCP21 is associated with GA-delayed leaf senescence, at least partly through the activation of the GA biosynthetic pathway. These findings expand our knowledge on the transcriptional mechanism of GA-mediated leaf senescence.

## 1. Introduction

Leaf senescence is a highly orchestrated biological process that is finely controlled by various intrinsic and external factors, such as developmental stage, natural plant hormones and stresses [1,2,3,4,5]. Generally, phytohormones including ethylene, abscisic acid (ABA), jasmonic acid (JA) and salicylic acid stimulate leaf senescence, whereas others such as cytokinins, auxins and polyamine inhibit senescence [1,5,6]. Gibberellins (GA), on the one hand, have been shown to delay leaf senescence of nasturtium [7], rumex [8], alstromeria [9] and herbaceous perennial *Paris polyphylla* [10], as well as delaying rose petal senescence [11]. On the other hand, exogenous GA_3_ treatment has been recently reported to promote senescence of Arabidopsis rosette leaves [12], indicating the complicated effects of GA on leaf senescence. Accordingly, it is important to elucidate the regulatory mechanisms of GA-mediated leaf senescence.

Increasing evidence demonstrates that transcription factors (TFs) are important regulatory proteins that control the onset and progression of leaf senescence by affecting the expression of senescence-associated genes (*SAGs*), hormone biosynthetic and signaling genes [5,6]. For example, Arabidopsis MYC2, 3 and 4 function redundantly to activate JA-induced leaf senescence by directly activating the expression of *SAG29* and chlorophyll catabolic genes (*CCGs*) [13]. In contrast, bHLH subgroup IIId TFs bHLH03, 13, 14 and 17, target the *SAG29* promoter and repress its expression [14]. NAM/ATAF/CUC (NAC) TFs of Arabidopsis [15], rice [16] and foxtail millet [17] function in ABA-induced leaf senescence by regulating ABA biosynthetic and signaling genes, or *CCGs*. A Chinese flowering cabbage ERF TF BrERF72 is found to enhance JA accumulation by inducing the expression of three JA biosynthetic genes (*BrLOX4*, *BrAOC3* and *BrOPR3*) during MeJA-promoted leaf senescence [18]. Interestingly, Arabidopsis WRKY45 is recently reported to be a key regulatory of GA transduction involved in the initiation of leaf senescence [12]. However, the transcriptional regulation of GA response in relation to leaf senescence is not clear.

TEOSINTE BRANCHED1/CYCLOIDEA/PCF (TCP) proteins, with the conserved TCP domains, are plant-specific TFs and have been identified in many plant species including Arabidopsis [19], rice [20], maize [21], tomato [22] and Chinese cabbage [23]. TCP TFs have been well documented to participate in multiple biological processes, such as cell proliferation and growth, and stress response [24]. Strikingly, several TCP TFs have been shown to not only mediate hormone-induced changes in cell proliferation, but also to act as modulators, or even key players, of hormone synthesis, transport and signal transduction [24]. For instance, Arabidopsis TCP15 is required for the correct balance between auxin levels and cytokinins responses in the developing carpel [25]. Rice OsTCP19 is strongly associated with abscisic acid (ABA)-mediated abiotic stress responses by binding and modulating the activity of OsABI4, overexpression of OsTCP19 in Arabidopsis affects not only ABA, but also auxin and JA signaling [26]. Recently, GhTCP19 from gladiolus is reported to be a positive regulator of the corm dormancy release process by the repression of an ABA biosynthesis gene expression (*GhNCED*), as well as the promotion of cytokinins biosynthesis (*GhIPT*) and signal transduction (*GhARR*) [27]. JA-related global responses are obviously affected by gain or loss of TCP activity [28], and three TCP TFs TCP20/TCP9 and TCP4, were further found to repress and activate the transcription of a JA biosynthetic gene *LOX2*, respectively, thus acting antagonistically in the control of JA-mediated leaf development and senescence [29]. Remarkably, several reports reveal that TCP proteins participate in various physiological processes controlled by GA [24]. Nevertheless, the molecular mechanisms underlying the link between TCP members and GA-mediated leaf senescence remain largely unknown. 

As one of the representatives of leafy vegetables, Chinese flowering cabbage (*Brassica rapa* ssp. *Parachinensis*), is popular in the diet of people in Asia due to its health-promoting compounds and valuable anticancer properties [18,30,31,32,33,34]. In general, flowering shoots, stems and younger leaves of this cabbage are harvested for eating. However, harvested cabbage leaves senesce rapidly during transportation and storage, becoming wilted and yellowed, which greatly reduces product quality and commercial value [18,30,31,32,33,34]. Thus, there is an imperative demand for the elucidation of the molecular mechanisms of leaf senescence of Chinese flowering cabbage. Such knowledge is of great value for developing strategies in practice to maintain the quality and to extend the shelf-life of this cabbages. In our previous studies, we identified and characterized several TFs including BrWRKY65 [30], BrWRKY6 [31], BrERF72 [18] and BrNAC055 [34] in association with leaf senescence in Chinese flowering cabbage, among which BrWRKY6 was shown to suppress the expression of two GA biosynthetic genes (*BrKAO2* and *BrGA20ox2*) during GA-delayed leaf senescence [31]. Despite these tremendous achievements, it is believed that more TFs are involved in leaf senescence, given the extensive complication of leaf senescence. The current study revealed that a TCP TF BrTCP21 was likely positively associated with GA-delayed leaf senescence by the direct activation of transcription of a GA biosynthetic gene *BrGA20ox3*. 

## 2. Results and Discussion

### 2.1. Exogenous GA_3_ Application Delays Leaf Senescence of Chinese Flowering Cabbage 

**Figure 1:**
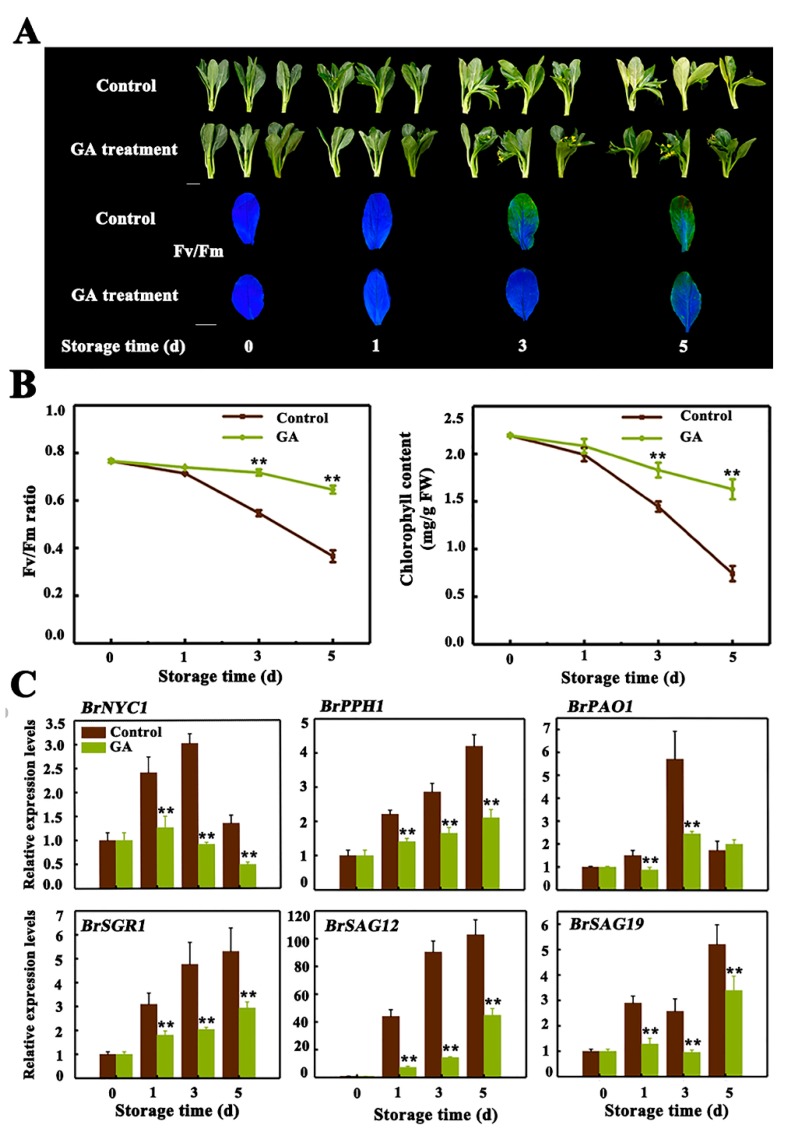
Gibberellin A3 (GA_3_) treatment delays leaf senescence of Chinese flowering cabbage. (**A**) Appearance and chlorophyll fluorescence imaging (Fv/Fm) of control and GA_3_-treated cabbage leaves during senescence. Bar = 5 cm.(**B**) Change of Fv/Fm and total chlorophyll content in control and GA_3_-treated cabbage leaves during senescence. (**C**) Relative expression of four *CCGs* (*BrNYC1*, *BrPPH1*, *BrPAO1* and *BrSGR1*) and two senescence-associated genes (*BrSAG12* and *BrSAG19*), in control and GA_3_-treated cabbage leaves during senescence. Data presented in (**B**) and (**C**) are the mean ± S.E. of three biological replicates. Asterisks indicate a significant difference in GA_3_-treated leaves compared with control leaves (Student’s *t*-test: * *p* < 0.05 and ** *p* < 0.01).

Previous studies indicate that the effect of GA on senescence seems contradictory, as GA treatment delays leaf senescence in nasturtium [7], rumex [8], alstromeria [9] and herbaceous perennial *Paris polyphylla* [10], while accelerates leaf senescence in Arabidopsis rosette leaves [12]. It has been suggested that the dosage of GA applied or the GA-treated leaves situation may have the opposite effect on leaf senescence [12,35]. Our previous study also showed that exogenous GA_3_ treatment delayed leaf senescence of Chinese flowering cabbage [31], which has been further verified in the present work. As shown in Figure 1A, appearance and noninvasive chlorophyll fluorescence imaging with maximum PSII quantum yield (Fv/Fm) default clearly demonstrates that in comparison to the control, GA_3_-treated cabbage leaves exhibited a reduced degree of yellowing symptom, on the third and fifth day of storage. As expected, the value of Fv/Fm and total chlorophyll content were significantly higher in GA_3_-treated leaves, which were about 1.76 and 2.20-fold of the control leaves respectively, at 5th day of storage (Figure 1B). To investigate why GA_3_ delayed leaf senescence, we quantified the relative abundance of transcripts of two *SAGs* (*BrSAG12* and *BrSAG19*) and four *CCGs* (*BrNYC1*, *BrPPH*, *BrPAO* and *BrSGR1*), after GA_3_ treatment. We found that the transcript levels of all these genes were dynamically repressed upon treatment with GA_3_, with a 2.32-, 0.73-, 1.33-, 1.34-, 5.26- and 1.68-fold repression for *BrNYC1*, *BrPPH*, *BrPAO*, *BrSGR1*, *BrSAG12* and *BrSAG19* at third day of storage, compared to that in the control, respectively (Figure 1C). 

### 2.2. BrTCP21 belongs to a Member of Class I TCP

It has been well documented that transcriptional regulation mediated by TFs such as MYCs, NACs, and WRKYs, plays an important role in leaf senescence modulated by various hormones [5,6]. Many TCP TFs have been identified in plants, however, only a few TCP members were reported to be involved in GA-controlled biological processes [24]. Thus, we focused on the identification of TCP TFs from our RNA-seq transcriptome database associated with Chinese flowering cabbage leaf senescence, and four genes annotated as TCP proteins were found to be down-regulated (unpublished data), among which member (GenBank number, XM_009124197.2) was obviously down-regulated during leaf senescence, and therefore captured our attention. 

**Figure 2:**
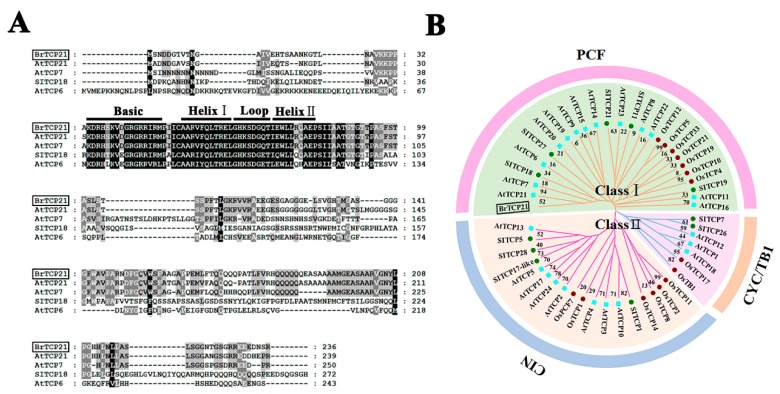
Multiple sequence alignment and phylogenetic analysis of BrTCP21. (**A**) Multiple alignment of BrTCP21 with other TEOSINTE BRANCHED1/CYCLOIDEA/PCF (TCP) members. The following proteins were used for analysis: AtTCP21 (NP_196450.1), AtTCP6 (XP_002870658.2), AtTCP7 (NP_197719.1) and SlTCP18 (ADM87267.1). Identical and similar amino acids are shaded in black and grey, respectively. Single underlining indicates the conserved non-canonical basic helix–loop–helix structure. (**B**) Phylogenetic analysis of TCPs. BrTCP21 was boxed. The phylogenetic tree was constructed with neighbor-joining test using MEGA program (version 5.0).

The resulting amplified full-length of this gene was 711 bp, encoding a protein with 236 amino acid residues, calculated molecular weight of 24.73 kDa, and *p*I value of 8.89. By investigating the NCBI database, it was found that this gene shares a high similarity of 92.9% to Arabidopsis AtTCP21, and it was therefore named BrTCP21. The most distinguished characteristic of TCP proteins is the presence of a ~59-amino-acid-long conserved region with a non-canonical basic helix–loop–helix (bHLH) structure (the so-called TCP domain) in their N-terminal, which has proven to be responsible for nuclear targeting, DNA binding and protein–protein interactions [23,36]. Multiple-alignment of BrTCP21 showed that, similar to other orthologous TCP proteins, BrTCP21 contained the conserved TCP domain (Figure 2A). On the basis of the TCP domain, the TCP members can be grouped into two distinct subfamilies: class I (PCF or TCP-P class) and class II (TCP-C class), among which class II can be further divided into subclades: CIN and CYC/TB1 [24,37]. As shown in Figure 2B, the phylogenetic tree displayed that BrTCP21 together with AtTCP21, AtTCP7 and SlTCP18, belong to class I. 

### 2.3. Molecular Characterization of BrTCP21

**Figure 3:**
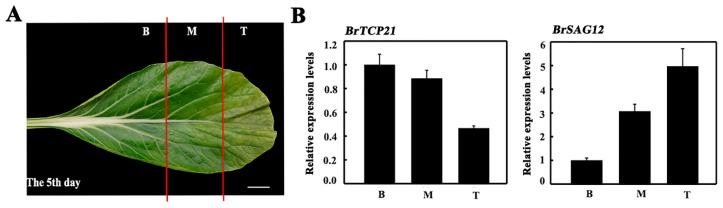
Localized expression of *BrTCP21* in a senescing leaf. (**A**) The third leaves from the bottom of cabbage plants on the 5th days was detached and separated into three parts as illustrated. *B* basal part, *M* middle part, *T* tip part. Bar = 2 cm. (**B**) Expression of *BrTCP21* and *BrSAG12*. Data are the mean ± S.E. of three biological replicates.

**Figure 4:**
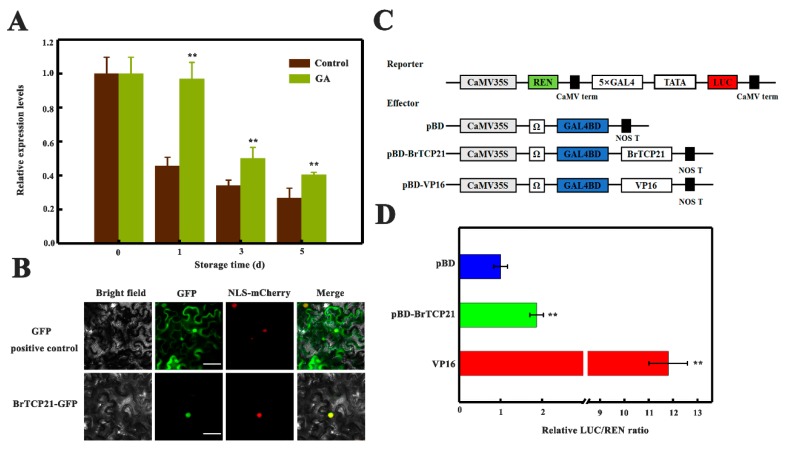
Molecular properties of BrTCP21. (**A**) Relative expression of BrTCP21 in control and GA_3_-treated cabbage leaves during senescence. Each value represents the mean ± S.E. of three biological replicates. Asterisks indicate a significant difference in GA_3_-treated leaves compared with control leaves (Student’s *t*-test: ** *p* < 0.01). (**B**) Subcellular localization of BrTCP21 in epidermal cells of *Nicotiana benthamiana* leaves. A plasmid harboring GFP or BrTCP21-GFP was transformed into *Nicotiana benthamiana* leaves by *Agrobacterium tumefaciens strain* EHA105. GFP signals was observed with a fluorescence microscope after 2 d of infiltration. NLS-mCherry was included in each transfection to serve as a control for nuclear localization. Bars, 30 μm. (**C**) Diagrams of the reporter and effector vectors. (**D**) Trans-activation of BrTCP21 in *Nicotiana benthamiana* leaves. The trans-activation ability of BrTCP21 was indicated by the ratio of luciferase (LUC) to renilla luciferase (REN). The LUC/REN ratio of the empty pBD vector (negative control) was used as a calibrator (set as 1). pBD-VP16 was used as a positive control. Data are means ± S.E. of six independent biological replicates. Asterisks represents significant differences at 0.01 level by Student’s *t*-test, compared to pBD.

To evaluate whether BrTCP21 is associated with GA-delayed leaf senescence, its expression pattern was investigated by quantitative real-time PCR (qRT-PCR). Cabbage leaf senesces serially from the tip to the base [31]. The representative third leaf from the bottom of the cabbage at the 5th day of storage was divided into three sections across the leaf axis (Figure 3A). Localized expression of *BrTCP21* in a senescing leaf showed that compared with the leaf basal section, the transcript level of *BrTCP21* was lower in the senescing middle and tip section, which was contrary to the expression of *BrSAG12* (Figure 3B). Consistent with the RNA-seq data, the transcript level of *BrTCP21* was decreased continuously during leaf senescence, while GA_3_ treatment inhibited the decline. In comparison with control leaves, the transcription level of *BrTCP21* was higher in GA_3_-treated leaves (Figure 4A). Generally, TCP proteins are localized to nuclei [36,38,39]. Subcellular localization prediction (WoLF PSORT online website, https://www.genscript.com/wolf-psort.html) also indicated that BrTCP21 was located in the nuclear region. To verify this prediction, BrTCP21 was fused to green fluorescent protein (GFP) and transiently expressed in *Nicotiana benthamiana* leaves. Compared with the fluorescence of control 35S-GFP that distributed throughout of the cell, the BrTCP21-GFP fusion protein, as the nuclear marker (NLS-mCherry), was exclusively detected in the nucleus (Figure 4B). To provide evidence for potential roles of BrTCP21 in transcriptional regulation, we assessed the transcriptional activity of BrTCP21 in vivo using the Dual-LUC reporter system. As shown in Figure 4C, similar with the activator control VP16, co-infiltration of BrTCP21 with the reporter significantly increased luciferase (LUC)/renilla luciferase (REN) ratio. These data reveal that BrTCP21 is a nuclear-localized transcriptional activator that is possibly associated with GA-delayed leaf senescence of Chinese flowering cabbage.

### 2.4. BrTCP21 Directly Binds to the Promoter of BrGA20ox3

**Figure 5:**
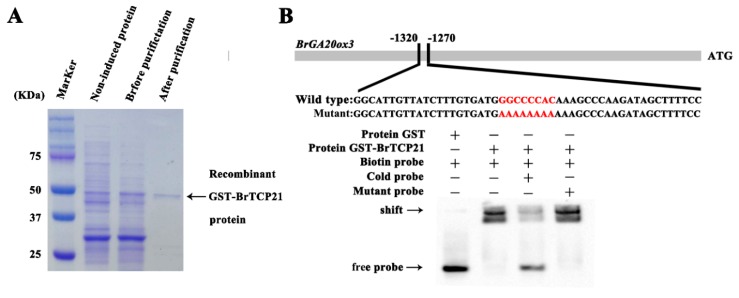
BrTCP21 directly binds to *BrGA20ox3* promoter. (**A**) SDS-PAGE gel stained with Coomassie brilliant blue demonstrating affinity purification of the recombinant GST-BrTCP21 protein used for electrophoretic mobility shift assay (EMSA). (**B**) EMSA showing the binding of BrTCP21 to the TCP binding site of the *BrGA20ox3* promoter. Purified glutathione-S-transferases (GST)-tagged BrTCP21 protein were incubated with the biotin-labeled wild-type probe containing the TCP binding site, and the DNA–protein complexes were separated on native polyacrylamide gels. Sequences of both the wild-type and mutated probes are shown at the top of the image (wild-type and mutated TCP binding site are marked with red letters). Shifted bands, suggesting the formation of DNA–protein complexes, are indicated by arrows. ‘‘−’’ represents absence, ‘‘+’’ represents presence. Competition experiments were carried out by adding cold probes with unlabeled DNA fragments with the same or mutant sequences.

We then intended to reveal the regulatory mechanism of BrTCP21 involved in GA-delayed leaf senescence of Chinese flowering cabbage. For this purpose, identifying the potential targets of BrTCP21 was essential. It is well-documented that the predicted consensus binding site of class I TCP TFs is GGNCCCAC, especially the core sequence GCCCR [24,29,36]. Noticeably, the ortholog TCP protein LANCEOLATE of tomato participates in GA biosynthesis by up-regulating the *SlGA20ox1* gene during leaf development [40]. However, whether LANCEOLATE can target the *SlGA20ox1* promoter directly is not confirmed. Similarly, with the expression of *BrTCP21*, the transcription level of three GA biosynthetic genes (*BrKAO2*, *BrGA20ox2* and *BrGA20ox3*) were maintained higher after GA_3_ treatment, which is associated with the suppression of leaf senescence by exogenous application of GA_3_ [31]. After surveying the promoter sequences of these GA biosynthetic genes, the TCP binding site was found only in *BrGA20ox3* promoter (Supplementary Text S1). To confirm the binding of BrTCP21 to *BrGA20ox3* promoter, an in vitro electrophoretic mobility shift assay (EMSA) was performed. Results showed that purified GST-BrTCP21 protein (Figure 5A), not the GST protein, bound to the TCP binding elements presented in *BrGA20ox3* promoter, causing mobility shift band (Figure 5B). Moreover, adding molar excesses of the unlabeled wild-type probes, but not the mutated fragments, eliminated the binding complex (Figure 5B), indicating the specificity of the DNA-protein interaction. 

### 2.5. BrTCP21 Activates the Transcription of BrGA20ox3

**Figure 6:**
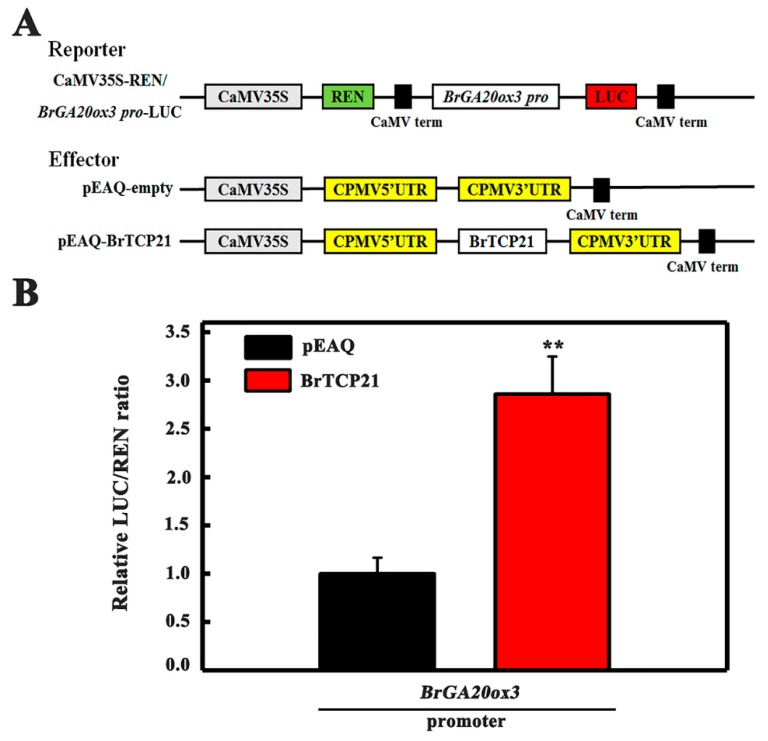
BrTCP21 enhances *BrGA20ox3* transcription by transient transcription dual-luciferase Dual-LUC assay in *Nicotiana benthamiana* leaves. (**A**) Diagrams of the reporter and effector vectors. (**B**) BrTCP21 activates *BrGA20ox3* promoter. Data are means ± S.E. of six independent biological replicates. Asterisks indicate significant differences by student’s *t-*test (** *p* < 0.01).

After determining *BrGA20ox3* as the potential downstream target of BrTCP21, and establishing BrTCP21 as a transcriptional activator, we were curious as to whether BrTCP21 could directly enhance the transcription of *BrGA20ox3*. To address this particular question, *Nicotiana benthamiana* leaves were co-transformed with transient over-expression effector construct containing 35S-BrTCP21, and a dual-luciferase reporter construct carrying *BrGA20ox3* promoter fused to LUC (Figure 6A). As illustrated in Figure 6B, expression of *BrTCP21* resulted in a more than 1.86-fold increase in *LUC* expression driven by *BrGA20ox3*, as compared to the vector control, providing evidence for a positive trans-activation activity of BrTCP21. Collectively, BrTCP21 binds directly to the promoter of *BrGA20ox3*, and activates its transcription, rendering it a direct target of BrTCP21 transcriptional regulation. 

TCP gene family has been identified in many plant species, with 24, 28 and 30 members in Arabidopsis [19], rice [20] and tomato [22] respectively. Recently, 39 *TCP* genes were identified on the whole genome of Chinese cabbage (*Brassica rapa* L. ssp. *pekinensis*), with 19 and 20 members belong to class I and II respectively [23]. The predicted consensus DNA-binding sites of the two different TCP classes are partly overlapping [24]. Interestingly, Arabidopsis class I (TCP9 and TCP20) and II (TCP4) TCP genes act antagonistically to control a JA biosynthetic gene *LOX2* expression through different regulatory motifs [29]. Similarly, whether class II TCP members can target *BrGA20ox3* to compete with BrTCP21’s regulation, needs to be elucidated. In addition, TCP TFs have important roles in various hormone biosynthesis and signaling pathways. For instance, a class I TCP TF GhTCP19 from gladiolus, has recently been reported to positively regulate corm dormancy release by suppressing the transcription of an ABA biosynthetic gene *GhNCED*, while activating the transcription of cytokinins biosynthetic gene *GhIPT* and signal transduction gene *GhARR* [27]. As ABA, JA and cytokinins have been demonstrated to accelerate and delay leaf senescence of Chinese flowering cabbage respectively [18,33,41], whether BrTCP21 or other TCP members such as GhTCP19 can exert dual regulatory roles in hormones-mediated leaf senescence, will be an interesting topic for consideration in the future. 

DNA-binding activity of TFs to their targets is strictly affected by many factors, such as homo-/hetero-dimerization, post-transcriptional/translational modification and protein–protein interactions [42]. It was discovered that in banana, MaTCP20 interacts with MaTCP5 or MaTCP19, forming complexes to co-regulate the transcriptions of cell wall-modifying genes during fruit ripening [43]. Additionally, DELLA proteins, the key repressors of GA responses, have been found to interact with different types of TFs including WRKY, bHLH and TCP, thereby inactivating their critical effects on plant development [24,44,45]. The first time protein–protein interactions between class I TCPs and DELLA proteins were reported in Arabidopsis by Davière et al. [46], who proposed a work model by which DELLA-TCP interactions controlled GA-determined plant height. In this model, DELLA binds to class I TCPs, in particular TCP14, and prevents TCP interaction with the promoter of targeted genes, notably cell cycle genes such as *CYCA2;3* and *CYCB1;1*. Thereafter, DELLA proteins were demonstrated to maintain the embryo in a dormant state by interacting with TCP14 and TCP15, and this interaction prevents TCP14 and TCP15 activity on cell proliferation in the embryonic root apical meristem [47]. Thus, elucidating whether BrTCP21-DELLA can form a complex to affect the expression of GA biosynthetic genes antagonistically, as well as identifying other interaction proteins and regulatory factors, will help provide more detailed information on the BrTCP21-mediated regulation of gene networks operating the GA-delayed leaf senescence of Chinese flowering cabbage. 

## 3. Materials and Methods 

### 3.1. Plant Materials, GA Treatment and Growth Conditions

Chinese flowering cabbages (*Brassica rapa* var. *parachinensis*) were planted in a local commercial vegetable farm near Guangzhou, southern China, with standard insecticide and fungicide management. Cabbages were harvested after 40 days of growth (March to April) and transported to laboratory under low temperature (4 °C) immediately. Uniform cabbages without appearance defects were selected and randomly divided into two groups for control and GA_3_ treatment respectively. GA_3_ (100 μM) and distilled water (control treatment) were foliar-sprayed on cabbages as described previously [31]. Subsequently, both control and GA-treated cabbages were stored at 15 °C in incubators for 5 days. On 0, 1, 3 and 5 days of storage, the third leaves from the bottom of 10 cabbages were collected as one replicate for physiological and molecular analysis. In addition, for evaluating localized expression of genes in a senescing leaf, the third leaves from the bottom of 10 control cabbages on the fifth day was detached, dissected into three sections, and sampled respectively. At least three biological replicates were used for all treatments in all experiments. All samples were immediately frozen in liquid nitrogen and stored at −80 °C for further assays. 

Tobacco (*Nicotiana benthamiana*) plants were planted in a growth chamber set at 22 °C and a 16-h photoperiod. Four-week-old tobacco plants were used for *Agrobacterium tumefaciens*-mediated transient expression assays. 

### 3.2. Physiological Measurements of Leaf Senescence

Leaf total chlorophyll content and Fv/Fm are physiological parameters commonly used as indicators of leaf senescence. Total chlorophyll content was measured by extracting approximately 0.1 g of leaves in 10 mL in 80% acetone in the dark for 24 h and measuring the absorbance of extracts at 663 nm and 645 nm using a spectrophotometer as described earlier [31]. Fv/Fm was determined noninvasively after leaves were adapted to dark conditions for 30 min through a chlorophyll fluorometer (Imaging-PAM-M series, Heinz Walz GmbH, Effeltrich, Germany) equipped with a charge-coupled device (CCD) camera that enabled the capture of high resolution digital images of the emitted fluorescence.

### 3.3. RNA Extraction, Gene Cloning and Bioinformatic Analysis

A Quick RNA Isolation Kit (Huayueyang, Beijing, China) was used to extract total RNA from cabbage leaves. RNA was then treated with DNase I to remove contaminating DNA and reverse transcribed to cDNA using the reverse transcriptase M-MLV (TaKaRa, Shiga, Japan) based on a 1 μg RNA template, following the manufacturer’s protocol. The full-length of *BrTCP21* gene was isolated from our transcriptome database. Theoretical isoelectric points (*pI*) and mass values were assessed on the website (http://web.expasy.org/compute_pi/). Sequence alignment and phylogenetic analysis of TCP proteins, based on a neighbor-joining method, were carried out with the CLUSTALW program (version 1.83). A phylogenetic tree was created with the MEGA5.0 program with neighbor-joining statistical method and 1000 bootstrap. 

### 3.4. Gene Expression via qRT-PCR

cDNAs from each sample were subjected to qRT-PCR assays. qRT-PCR was performed using the step one plus a CFX96 Touch™ real-time PCR detection system (Bio-Rad, Hercules, CA, USA) and GoTaq qPCR master mix kit (Promega, Madison, WI, USA). The cycling began with an initial denaturation step at 94 °C for 5 min, followed by 40 cycles of 94 °C for 10 s, 60 °C for 30 s, and 72 °C for 30 s. A no-template control and melting curve analysis was included in every PCR run. The expression levels of target genes were normalized according to the cycle threshold (*C*t) value using *BrActin1* [48] as the reference gene. 

### 3.5. Subcellular Localization Assay

The BrTCP21 coding sequence (CDS) fragment (without the stop codon) was amplified and inserted into the pEAQ-GFP vector to produce the fusion protein BrTCP21-GFP. Then BrTCP21-GFP and the control pEAQ-GFP constructs were transformed to *Agrobacterium tumefaciens* strain EHA105. Overnight cultures of *Agrobacteria* were collected by centrifugation, resuspended in MES buffer to 0.8–1.0 OD_600_, incubated at room temperature for 2 h before infiltration. *Agrobacteria* suspension in a 1-mL syringe (without the metal needle) was carefully press-infiltrated manually onto healthy leaves of 4-week-old *Nicotiana benthamiana* as described previously [49]. After 2 days of infiltration, the GFP fluorescent signals in the epidermal cells of leaves were directly observed and images were captured by using a Zeiss fluorescence microscope. 

### 3.6. Recombinant Protein Induction, Purification and EMSA Assay

For protein expression and purification, the BrTCP21 coding sequence was recombined into the vector pGEX-4T-1, and transformed into *Escherichia coli* strain Transetta (DE3). When cell density reached 0.6 (OD_600_), protein expression in 1-L cultures was induced at 37 °C by addition of 0.3 mM isopropyl thio-β-D-galactoside in the course of 3 h. Cells were harvested by centrifugation. The cell pellet was resuspended in phosphate-buffered saline and incubated on ice for 30 min. Following sonication, the lysate was cleared by centrifugation. Glutathione-Superflow Resin (Clontech, Mountain View, CA, USA) was used for purification of GST-tagged protein by gravity-flow chromatography according to the protocol of the manufacturer, followed by SDS-PAGE and Coomassie Brilliant Blue staining to confirm protein size and purity.

Electrophoretic mobility shift assay (EMSA) was performed as described previously [50,51]. The synthetic nucleotides (~60 bp) derived from the 5′ UTR of *BrGA20ox3* oligonucleotides, which contain the consensus binding site (GGNCCCAC) of TCPs, were biotin-labeled at the 5′ end. The purified recombinant BrTCP21 was incubated with biotin-labeled probes in binding buffer for 25 min at 30 °C. Competitions were carried out by adding cold probes with unlabeled DNA fragments with the same or mutant sequences. The reaction mixture was electrophoresed on a 6% native polyacrylamide gels, and then transferred onto a positively charged nylon membrane, followed by cross-linking though illumination under an ultraviolet lamp. The signals from the labeled DNA were detected by using the LightShift chemiluminescent EMSA kit (Thermo Scientific, Rockford, IL, USA) in a ChemiDoc™ MP Imaging System (Bio-Rad).

### 3.7. Transient Transcription Dual-Luciferase (Dual-LUC) Assays

To investigate the transcriptional ability of BrTCP21 in vivo, its full length was inserted into pBD to construct pBD-BrTCP21 as effector. The positive control (pBD-VP16) was constructed by fusing VP16, a herpes simplex virus-encoded transcriptional activator, to pBD. pBD itself was used as a negative control. The GAL4 plasmid with *firefly luciferase* (*LUC*) gene was used as a reporter, and the *renilla luciferase* (*REN*) gene in the same plasmid was used as an internal control.

To determine the activation of *BrGA20ox3* by BrTCP21, the promoter fragments of *BrGA20ox3* was amplified and cloned into the transient dual luciferase expression vector pGreenII 0800-LUC as reporter constructs. To generate 35S::BrTCP21 effector construct, the BrTCP21 coding sequence were amplified by PCR and inserted into pEAQ vector. The empty vector was included as a control.

Transient transcription Dual-LUC assays were performed using *Nicotiana benthamiana* plants as described [31,51]. The reporter and effector constructs mentioned above were co-infiltrated into tobacco leaves. After 2 days of infiltration, the luciferase activity of tobacco leaf extract was quantified by a Luminoskan Ascent Microplate Luminometer (Thermo Fisher Scientific, Rockford, IL, USA), using commercial dual-luciferase reporter assay kit according to the manufacturer’s instruction (Promega). The trans-activation ability of BrTCP21 was indicated by the LUC/REN ratio.

### 3.8. Statistical Analysis

Data are represented as means ± S.E. of three or six biological repeats. Statistical differences of two treatments were performed by applying Student’s *t*-test. Data are considered significant as follows: * *p* < 0.05, ** *p* < 0.01.

### 3.9. Primers

All primers used in this research are listed in Supplementary Table S1. 

## 4. Conclusions

Taken together, exogenous GA_3_ treatment delays leaf senescence of Chinese flowering cabbage. A class I TCP member BrTCP21, which is GA-responsive and acts as a nuclear-localized transcriptional activator, is identified. Moreover, BrTCP21 targets the promoter of a GA biosynthetic gene *BrGA20ox3*, leading to the activation of its transcription. These findings expand our understanding of TCP TFs’ functions and shed light on the transcriptional regulatory mechanism operating GA-mediated leaf senescence, thereby laying the foundation for exploring new effective techniques to maintain the postharvest quality of leafy vegetables.

## Supplementary Materials

The following are available online at https://www.mdpi.com/1422-0067/20/16/3860/s1.

## References

[1] Jibran R., Hunter D., Dijkwel P. (2013). Hormonal regulation of leaf senescence through integration of developmental and stress signals. Plant Mol. Biol..

[2] Schippers J.H., Schmidt R., Wagstaff C., Jing H.C. (2015). Living to die and dying to live: The survival strategy behind leaf senescence. Plant Physiol..

[3] Schippers J.H. (2015). Transcriptional networks in leaf senescence. Curr. Opin. Plant Biol..

[4] Kim J., Woo H.R., Nam H.G. (2016). Toward systems understanding of leaf senescence: An integrated multi-omics perspective on leaf senescence research. Mol. Plant.

[5] Woo H.R., Kim H.J., Lim P.O., Nam H.G. (2019). Leaf senescence: Systems and dynamics aspects. Annu. Rev. Plant Biol..

[6] Jan S., Abbas N., Ashraf M., Ahmad P. (2019). Roles of potential plant hormones and transcription factors in controlling leaf senescence and drought tolerance. Protoplasma.

[7] Beevers L. (1966). Effect of gibberellic acid on the senescence of leaf discs of Nasturtium (*Tropaeolum majus*). Plant Physiol..

[8] Whyte P., Luckwill L.C. (1966). A sensitive bioassay for gibberellins based on retardation of leaf senescence in *Rumex obtusifolius* (L.). Nature.

[9] Kappers I.F., Jordi W., Maas F.M., Stoopen G.M., van der Plas L.H.W. (1998). Gibberellin and phytochrome control senescence in Alstromeria leaves independently. Physiol. Plant..

[10] Yu K., Wei J., Ma Q., Yu D., Li J. (2009). Senescence of aerial parts is impeded by exogenous gibberellic acid in herbaceous perennial *Paris polyphylla*. J. Plant Physiol..

[11] Lü P., Zhang C., Liu J., Liu X., Jiang G., Jiang X., Khan M.A., Wang L., Hong B., Gao J. (2014). RhHB1 mediates the antagonism of gibberellins to ABA and ethylene during rose (*Rosa hybrida*) petal senescence. Plant J..

[12] Chen L., Xiang S., Chen Y., Li D., Yu D. (2017). Arabidopsis WRKY45 interacts with the DELLA protein RGL1 to positively regulate age-triggered leaf senescence. Mol. Plant.

[13] Zhu X., Chen J., Xie Z., Gao J., Ren G., Gao S., Zhou X., Kuai B. (2015). Jasmonic acid promotes degreening via MYC2/3/4- and ANAC019/055/072-mediated regulation of major chlorophyll catabolic genes. Plant J..

[14] Qi T., Wang J., Huang H., Liu B., Gao H., Liu Y., Song S., Xie D. (2015). Regulation of jasmonate-induced leaf senescence by sntagonism between bHLH subgroup IIIe and IIId factors in Arabidopsis. Plant Cell.

[15] Yang J., Worley E., Udvardi M. (2014). A NAP-AAO3 regulatory module promotes chlorophyll degradation via ABA biosynthesis in Arabidopsis leaves. Plant Cell.

[16] Liang C., Wang Y., Zhu Y., Tang J., Hu B., Liu L., Ou S., Wu H., Sun X., Chu J. (2014). OsNAP connects abscisic acid and leaf senescence by fine-tuning abscisic acid biosynthesis and directly targeting senescence-associated genes in rice. Proc. Natl. Acad. Sci. USA.

[17] Ren T., Wang J., Zhao M., Gong X., Wang S., Wang G., Zhou C. (2018). Involvement of NAC transcription factor SiNAC1 in a positive feedback loop via ABA biosynthesis and leaf senescence in foxtail millet. Planta.

[18] Tan X.L., Fan Z.Q., Shan W., Yin X.R., Kuang J.F., Lu W.J., Chen J.Y. (2018). Association of BrERF72 with methyl jasmonate-induced leaf senescence of Chinese flowering cabbage through activating JA biosynthesis-related genes. Hortic. Res..

[19] Cubas P., Lauter N., Doebley J., Coen E. (1999). The TCP domain: A motif found in proteins regulating plant growth and development. Plant J. Cell Mol. Biol..

[20] Kosugi S., Ohashi Y. (1997). PCF1 and PCF2 specifically bind to cis elements in the rice proliferating cell nuclear antigen gene. Plant Cell.

[21] Doebley J., Stec A., Hubbard L. (1997). The evolution of apical dominance in maize. Nature.

[22] Parapunova V., Busscher M., Busscher-Lange J., Lammers M., Karlova R., Bovy A.G., Angenent G.C., de Maagd R.A. (2014). Identification, cloning and characterization of the tomato TCP transcription factor family. BMC Plant Biol..

[23] Liu Y., Guan X., Liu S., Yang M., Ren J., Guo M., Huang Z., Zhang Y. (2018). Genome-wide identification and analysis of TCP transcription factors involved in the formation of leafy head in Chinese cabbage. Int. J. Mol. Sci..

[24] Nicolas M., Cubas P. (2016). TCP factors: New kids on the signaling block. Curr. Opin. Plant Biol..

[25] Lucero L.E., Uberti-Manassero N.G., Arce A.L., Colombatti F., Alemano S.G., Gonzalez D.H. (2015). TCP15 modulates cytokinin and auxin responses during gynoecium development in Arabidopsis. Plant J..

[26] Mukhopadhyay P., Tyagi A.K. (2015). OsTCP19 influences developmental and abiotic stress signaling by modulating ABI4-mediated pathways. Sci. Rep..

[27] Wu J., Wu W., Liang J., Jin Y., Gazzarrini S., He J., Yi M. (2019). GhTCP19 transcription factor regulates corm dormancy release by repressing *GhNCED* expression in gladiolus. Plant Cell Physiol..

[28] Schommer C., Palatnik J.F., Aggarwal P., Chételat A., Cubas P., Farmer E.E., Nath U., Weigel D. (2008). Control of jasmonate biosynthesis and senescence by miR319 targets. PLoS Biol..

[29] Danisman S., van der Wal F., Dhondt S., Waites R., de Folter S., Bimbo A., van Dijk A., Muino J., Cutri L., Dornelas M. (2012). Arabidopsis class I and class II TCP transcription factors regulate jasmonic acid metabolism and leaf development antagonistically. Plant Physiol..

[30] Fan Z.Q., Tan X.L., Shan W., Kuang J.F., Lu W.J., Chen J.Y. (2017). BrWRKY65, a WRKY transcription factor, is involved in regulating three leaf senescence-associated genes in Chinese flowering cabbage. Int. J. Mol. Sci..

[31] Fan Z.Q., Tan X.L., Shan W., Kuang J.F., Lu W.J., Chen J.Y. (2018). Characterization of a transcriptional regulator, BrWRKY6, associated with gibberellin-suppressed leaf senescence of Chinese flowering cabbage. J. Agric. Food Chem..

[32] Ombra M.N., Cozzolino A., Nazzaro F., d’Acierno A., Tremonte P., Coppola R., Fratianni F. (2017). Biochemical and biological characterization of two Brassicaceae after their commercial expiry date. Food Chem..

[33] Zhang X., Zhang Z., Li J., Wu L., Guo J., Ouyang L., Xia Y., Huang X., Pang X. (2011). Correlation of leaf senescence and gene expression/activities of chlorophyll degradation enzymes in harvested Chinese flowering cabbage (*Brassica rapa* var. *parachinensis*). J. Plant Physiol..

[34] Fan Z.Q., Tan X.L., Chen J.W., Liu Z.L., Kuang J.F., Lu W.J., Shan W., Chen J.Y. (2018). BrNAC055, a novel transcriptional activator, regulates leaf senescence in Chinese flowering cabbage by modulating reactive oxygen species production and chlorophyll degradation. J. Agric. Food Chem..

[35] Chen M., Maodzeka A., Zhou L., Ali E., Wang Z., Jiang L. (2014). Removal of DELLA repression promotes leaf senescence in Arabidopsis. Plant Sci..

[36] Zheng X., Yang J., Lou T., Zhang J., Yu W., Wen C. (2019). Transcriptome profile analysis reveals that CsTCP14 induces susceptibility to foliage diseases in cucumber. Int. J. Mol. Sci..

[37] Martin-Trillo M., Cubas P. (2010). TCP genes: A family snapshot ten years later. Trends Plant Sci..

[38] Du J., Hu S., Yu Q., Wang C., Yang Y., Sun H., Yang Y., Sun X. (2017). Genome-wide identification and characterization of BrrTCP transcription factors in *Brassica rapa* ssp. rapa. Front. Plant Sci..

[39] Fan H.M., Sun C.H., Wen L.Z., Liu B.W., Ren H., Sun X., Ma F.F., Zheng C.S. (2019). CmTCP20 plays a key role in nitrate and auxin signaling-regulated lateral root development in *Chrysanthemum*. Plant Cell Physiol..

[40] Yanai O., Shani E., Russ D., Ori N. (2011). Gibberellin partly mediates LANCEOLATE activity in tomato. Plant J..

[41] Tan X.L., Fan Z.Q., Kuang J.F., Lu W.J., Reiter R.J., Lakshmanan P., Su X.G., Zhou J., Chen J.Y., Shan W. (2019). Melatonin delays leaf senescence of Chinese flowering cabbage by suppressing ABFs-mediated abscisic acid biosynthesis and chlorophyll degradation. J. Pineal Res..

[42] Shaikhali J. (2015). GIP1 protein is a novel cofactor that regulates DNA-binding affinity of redox-regulated members of bZIP transcription factors involved in the early stages of Arabidopsis development. Protoplasma.

[43] Song C.B., Shan W., Yang Y.Y., Tan X.L., Fan Z.Q., Chen J.Y., Lu W.J., Kuang J.F. (2018). Heterodimerization of MaTCP proteins modulates the transcription of MaXTH10/11 genes during banana fruit ripening. BBA-Gene Regul. Mech..

[44] Xu H., Liu Q., Yao T., Fu X. (2014). Shedding light on integrative GA signaling. Curr. Opin. Plant Biol..

[45] Davière J.M., Achard P. (2016). A pivotal role of DELLAs in regulating multiple hormone signals. Mol. Plant.

[46] Davière J.M., Wild M., Regnault T., Baumberger N., Eisler H., Genschik P., Achard P. (2014). Class I TCP-DELLA interactions in inflorescence shoot apex determine plant height. Curr. Biol..

[47] Resentini F., Felipo-Benavent A., Colombo L., Blázquez M.A., Alabadì D., Masiero S. (2015). TCP14 and TCP15 mediate the promotion of seed germination by gibberellins in *Arabidopsis thaliana*. Mol. Plant.

[48] Qi J.N., Yu S.C., Zhang F.L., Shen X.Q., Zhang X.Z., Yu Y.J., Zhang D.S. (2010). Reference gene selection for real-time quantitative polymerase chain reaction of mRNA transcript levels in Chinese Cabbage (*Brassica rapa L*. ssp. pekinensis). Plant Mol. Biol. Rep..

[49] Wei W., Cheng M.N., Ba L.J., Zeng R.X., Luo D.L., Qin Y.H., Liu Z.L., Kuang J.F., Lu W.J., Chen J.Y. (2019). Pitaya HpWRKY3 is associated with fruit sugar accumulation by transcriptionally modulating sucrose metabolic genes *HpINV2* and *HpSuSy1*. Int. J. Mol. Sci..

[50] Fan Z.Q., Ba L.J., Shan W., Xiao Y.Y., Lu W.J., Kuang J.F., Chen J.Y. (2018). A banana R2R3-MYB transcription factor MaMYB3 is involved in fruit ripening through modulation of starch degradation by repressing starch degradation-related genes and *MabHLH6*. Plant J..

[51] Cheng M.N., Huang Z.J., Hua Q.Z., Shan W., Kuang J.F., Lu W.J., Qin Y.H., Chen J.Y. (2017). The WRKY transcription factor HpWRKY44 regulates *CytP450-like1* expression in red pitaya fruit (*Hylocereus polyrhizus*). Hortic. Res..

