# Supplementary material for: Activation of the Transcription of BrGA20ox3 by a BrTCP21 Transcription Factor Is Associated with Gibberellin-Delayed Leaf Senescence in Chinese Flowering Cabbage during Storage
Source: Int J Mol Sci. 2019 Aug 8;20(16):3860. doi: 10.3390/ijms20163860 (PMC6720506; doi:10.3390/ijms20163860)
Supplement: Supplementary file 1 [file ijms-20-03860-s001.pdf]

Supplementary materials

Table S1. List of primers used in this study.

Assay	primer sequence			Restriction Site
		Forward primer(5'-3')	Reverse primer(5'-3')	
RT-qPCR	***BrActin1***	CGCTTAACCCGAAAGCTAAC	TACGCCCACTAGCGTAAAG	
	BrTCP21	AGGAACTCTTAACGCCGTGA	GAGCTGCGCATATGATAGGC	
	BrNYC1	CGGAGACGGTGGCTAGAACG	TGCTTCTCCTGAGCCACGAC	
	BrPPH1	TATCTGATGCGCGGGTGGAT	TTCCCGACCAATGCTGGACT	
	BrPAO1	CAGCTTCAGCGACACTCACC	TCGCCGTGCTCTTCTTCGAT	
	BrSGR1	GTTGGGGTCCGCTTTGGGAA	AATCGAGCTAACCTGCGGGA	
	BrSAG12	CACTGGCGGCTTAACCACTGAA	GAAGATTGGCTGTATCCTACGGC	
	BrSAG19	GCAAGCGAGCGTTGGTAAAGGT	GGGTTGATTCTTCCACTCCCTTC	
**Subcellular localization**	***BrTCP21-pBEGFP-For***	***ATCTAGAGCAGTCGACGGTACCATGTCGAACGACGACGGGA***		***KpnI***
	BrTCP21-pBEGFP-Rev	***CTCCTCGCCCTTGCTCACCATACGTGAGTTATCCTCCTCCTC***		***BamHI***
EMSA	***pGEX-4T-1-BrTCP21-For***	GGTTCCGCGTGGATCCATGTCGAACGACGACGGGA		***BamHI***
	pGEX-4T-1-BrTCP21-Rev	AGTCACGATGCGGCCGC TCAACGTGAGTTATCCTCCTC		***NotI***
	EMSA –BrGA20ox3 pro -For	GGCATTGTTATCTTTGTGATGGGCCCCACAAAGCCCAAGATAGCTTTTCC		
	EMSA-BrGA20ox3pro-Rev	GGAAAAGCTATCTTGGGCTTTGTGGGGCCCATCACAAAGATAACAATGCC		
**Dual LUC assay**	***pBD-BrTCP21-For***	*TCGCCGACCGGTAGGCCTATGTCGAACGACGACGGGA*		***Stu I***
	pBD-BrTCP21-Rev	*AACCAGAGTTAAAGGCCTTCAACGTGAGTTATCCTCCTC*		***Stu I***
	pEAQ-BrTCP21-For	CAAATTCGCGACCGGT ATGTCGAACGACGACGGGA		***AgeI***
	pEAQ-BrTCP21-Rev	AGTTAAAGGCCTCGAG TCAACGTGAGTTATCCTCCTC		***XhoI***
	0800-BrGA20ox3-For	TATAGGGCGAATTGGGTACCATTGGCAGACAGAGACATGACG		***KpnI***
	0800- BrGA20ox3--Rev	TTGGCGTCTTCCATGGTTATCTTTATTTGTATATATATATATATATAGTAG		***NcoI***

Text S1. Promoter nucleotide sequences of *BrGA20ox3*. GGCCCCAC is marked by red and boxed. Translation start site (ATG) was shown in yellow box.

BrGA20ox3 (XM_009124115.2) promoter

ATTGGCAGACAGAGACATGACGACCTATTACAAAAGACCAAATCGATATGCTCGAGGATGGAATCTTGTATACTAAAGA

AAACGTTACTTTAAATTGGTTCTGCTGTTTCTATACATCCAAAATTGATATTATGTAGTTGCCAATTTAGATGGGTTTTTAC

ATAGCTCCTTCTCAGCAATAAAAGGATGAAACGTTCGGTTCTTTAACACCAAGAATCTGTTTTTTTCATACTTTAGCTTTA

ATAACCAAGAAAAAGCCAACTTATCTTTAAATTACGTATCTGATTTTGATCAATCAGCTAATACCAATCGGCATTGTTATCT

TTGTGATGGGCCCCACAAAGCCCAAGATAGCTTTTCCTGATGACTGCTAGTGGTTTCATTGAAACAACGAAACGGCCAC

GATTTAGAAAGAACATTTTTTTTTCGAAACAGTCGATGAACTCTGTAACGGACGATTGTTGTGTTGTTGTGGGTAGGTTC

GAGTGTTTTGAGAAAATAAGATCCAAAATAAGAGCATGGAAGACAAAAAGAGCCATATTCTGACTCCGGCTCTGATTGA

GCAGCCGAAGTAAGTAGCGACGGAAGCAACTATAAGAATTAAAGAGAAGGCAGAGAAAATGAATTCCTATTTTTTAAA

GATTAGAATACTTCCTTAGACTATTCTCTACTTTTTCTTTTCTTTTTGAAAAATGACATTCAAATTAAAAACATAAAACATA

CAAGGTATGGTTTTGAAACCATAAAGTTTTACAAAGTATAATGAGAAACAATCCTCATATTGAACTAAAGAATAACGTAA

AAAATAGAAACTCATGGACATAAGCTTAATAAAGCAAGATAAGAAAGAAGGAAACACTAGGGAGTGTGGTATTCTCGG

CCTCGACGACCTTTCTCTCGTATTTGGTCCAATCCATATCTTGGTATTTTTGACTTGGTTTTATTGATCTTTCACCTCATGA

CATGTTGAGATTTGCGGTAGCATCACTCATTAACCCATCTCCAACATCATGAACATTGAATTGCTCAATATCATTGTGAAA

AGGGCTGCCATGCGTAGGTGTGTACATAAATGCCAATGAATTGCTTCCTGATTCCAAACTGGTCTAGGAAACAATAATAT

GAGATGGAATTTCTTCCATAATAGTTGGTTCAGCTGGAGCAGTACCTGCTAATGGTGATGTAGTGAATTGAGAGTTGGGG

CCAATGGCTACTGTTCTCTACTTTTCCAAATTAACGAAACTGTTAACCTTTTTTTTTTAAACACTGATGATTCTATTAAAAC

ATGAAACAGACTACAAGAGCGGGGCGATGAACGATCTTGGAATACAACGTCCAGCGGACAACAACACAAAGTCCCAC

CAAAATTAAGCCCACAAGTGGGAGACTACATGAAAGACGTACATAAACGACAAACACTGACCCAAAAATATAATTTATA

AAAAAATAAACAAAACACACAAACACTGACGTCACTATTCCATACGCCTAAGATTTCCTCTCACCCCCTATAAATAGCAC

TCCTTTCTCCAGTTACATGACCACTATATTTATTTATATACCTCAGAAGCCCTTTAACGGATCTTTCATTTCCACTACTATAT

ATATATATATATACAAATAAAGATAAATG
